# Tunable Magneto-Optical Kerr Effects of Nanoporous Thin Films

**DOI:** 10.1038/s41598-017-03241-7

**Published:** 2017-06-06

**Authors:** Weiwei Zhang, Jianjun Li, Xiaokun Ding, Philippe Pernod, Nicolas Tiercelin, Yujun Song

**Affiliations:** 1 Department of Applied Physics, Center for Modern Physics Technology, Beijing Key Laboratory for Magneto-Photoelectrical Composite and Interface Science, University of Science and Technology Beijing, Beijing, 100083 China; 2 Univ. Lille, CNRS, Centrale Lille, ISEN, Univ. Valenciennes, UMR 8520-IEMN-LIA LICS, F-59000 Lille, France

## Abstract

Magnetoplasmonics, combining magnetic and plasmonic functions, has attracted increasing attention owing to its unique magnetic and optical properties in various nano-architectures. In this work, Ag, CoFeB and ITO layers are fabricated on anodic aluminum oxide (AAO) porous films to form hybrid multi-layered nanoporous thin films by magnetron sputtering deposition process. The designed nanostructure supports localized surface plasmon resonance (LSPR) and tunable magneto-optical (MO) activity, namely, the sign inversion, which can be controlled by AAO porous film geometry (pore diameter and inter-pore spacing) flexibly. The physical mechanism of this special MO phenomena is further analyzed and discussed by the correlation of Kerr rotation and electronic oscillations controlled by the surface plasmon resonance that is related to the nanoporous structure.

## Introduction

Plasmonics^1, 2^ preserves varieties of applications in high dimentional data storage^3–8^, high sensitive chemical detection^9–13^, biosensing^14–19^ and so forth^20^. This is mainly attributed to the ability of surface plasmons to conduct the subdiffraction-limit of light, to enhance the local surface electromagnetic fields^21^ or to allow localization of light at nanoscale dimensions^22^. It has been reported that plasmonic properties of metal nanoparticles intrinsically rely on their size, shape, surface topography, crystal structure, inter-particle spacing and dielectric environment^23, 24^. One development in plasmonics is magnetoplasmonics. Magnetoplasmonics promotes great interests on the momentum in photonics and magnetism sectors that are concerned with the resonant enhancement of light-magnetic-matter interaction^25^ with the rapid development of nanofabrication techniques^26^ (e.g., nanoimprint^27, 28^, lithography^29–35^, physical vapor deposition^36, 37^ and microfluidic synthesis process^38–40^). One topic in magnetoplasmonics is the enhancement of the magneto-optical effects in plasmonic nanostructures^41^. Magnetoplasmonics in nanostructures has potential to provide the flexibility in the reception and emission of photon and the light control at nanoscale, which are crucial in lots of emergent nano-optical applications^42^. For instance, when an incident light beam is well coupled to surface plasmon polartions inside the noble metal film (e.g., Ag^43–45^ and Au^46–48^) that has been deposited on magnetic components (e.g., Co, CoFeB, CoPt and NiO) and then reflected back to the optical field, its properties (e.g., intensity and the polarization status) become much sensitive to the medium permittivity and magnetization^49^.

The polarization status of light, used as information carriers, not only has great potential in biochemosensing^50^, optical communications and ultra-sensitive imaging^51^, but also plays important roles in the photonic transfer of quantum information^52, 53^. A coupled model of incoming electromagnetic excitation and the collective oscillation of free-electrons near the surface of metallic nanostructures has attracted more and more attention owing to their enhanced plamonic and magneto-optical properties^54^, such as enhanced Raman scattering^55, 56^, tunable nonlinear optical effects, surface plasmon polariton (SPP) and magneto-optical (MO) effects (i.e., Zeeman, Faraday or Kerr effects)^37, 57–62^.

Anomalous magneto-optical Kerr effects (MOKE) phenomena have been observed in various nanostructures^36, 50, 63, 64^. Localized surface plasmon resonance (LSPR) can be exploited in controlled manipulation of the MO response of nanostructured ferromagnetic nickel nanodisks, in which the inversed Kerr rotation is observed^50^. Calculations of bi-layered perforated nanostructure films formed by a gold layer and a smooth iron garnet layer show much higher transverse MOKE than the bare garnet film^57^. Hexagonally arrayed ferromagnetic nanowire films exhibit an enhanced Kerr rotation, which shows a strong dependence on nanowire diameters^65^. Optical and MO properties of hexagonally arrayed ferromagnetic nanoporous films show a complex MO spectrum with a much higher polarization rotation than that of the pure Co film^66, 67^. And some other systems accompanied by the enhanced MO response like Au/Co/Au nanosandwiches^68, 69^, gold-coated maghemite nanoparticles^70^, ferromagnetic garnet films incorporating Au nanoparticles^71^, Co@Ag core-shell nanoparticles^72^ and Co/Pt multi-layers deposited on polystyrene sphere arrays^73^ are also reported to have unique localized and/or propagating resonant excitations^50^. However, little attention has been paid on the nanoporous films consisting of noble metals, dielectrics and magnetic materials, which support strong LSPR and special MO response.

Anodic aluminum oxide (AAO) porous film, long-range ordered self-organized^74^ hexagonal columnar cells with central, cylindrical, uniform size holes, can be fabricated by the traditional 2-step anodizing process^23, 75, 76^ economically. This special nanoporous structure is assigned to the mechanical stress at the aluminum/alumina interface. This is proposed to cause repulsive forces between the neighboring pores^75^. The porous film is a useful template in the fabrication of devices^76^ (e.g., photoelectronic device^77^ and nanoparticle assemblies^78^ whose interfacial interaction can be regulated by the AAO structure, such as pore size, membrane thickness and surface morphologies) and various functional nanostructures^79–82^ (e.g., solar cells^83^, nanotubes^84–86^, nanofibers^86^, catalysts^87–89^, and metal nanowires^90^). To date, the AAO porous film also has a wide applications in ultrafiltration^91^, biosensors^92–94^, photonics^95, 96^, masking^97^ and information storage^98^. Many types of nanocomposites have been fabricated on the AAO porous film by magnetron sputtering deposition process and the deposited thin films can form a densely packed, regular and almost petal like structure around the pores^99^. This special structure permits an opportunity to realize LSPR, to enhance Fabry-Pérot interferences^11^ and to tailor magneto-optical Kerr effects.

In this work, a porous nanostructure with intertwined plasmonic and magnetic properties is developed via depositing Ag, CoFeB and ITO layers on morphology controlled AAO porous film to form hybrid multi-layered nanoporous films by a magnetron sputtering deposition process. From the analysis of the experimental data, the nanoporous structure supports localized surface plasmon resonance and tunable inversion of MOKE hysteresis loops.

## Results

### Morphology and Composition

To study the effect of the AAO porous film geometry on the surface plasmon activity and MOKE inversion, the inter-pore spacing of two AAO substrates was first set to two different values (i.e., 110 nm and 450 nm). Then, the pore diameter of three samples with the same inter-pore spacing 110 nm was set to 40 nm, 60 nm and 100 nm. Similarly, the pore diameter of three samples with the same inter-pore spacing 450 nm was set to 130 nm, 160 nm and 200 nm. Finally, hybrid multi-layered films Ag (5 nm)/ITO (10 nm)/CoFeB (10 nm)/ITO (10 nm)/Ag (5 nm) were fabricated on the prepared AAO templates. Thus, six hybrid multi-layered samples with different nanoporous geometries were fabricated and characterized.

**Figure 1:**
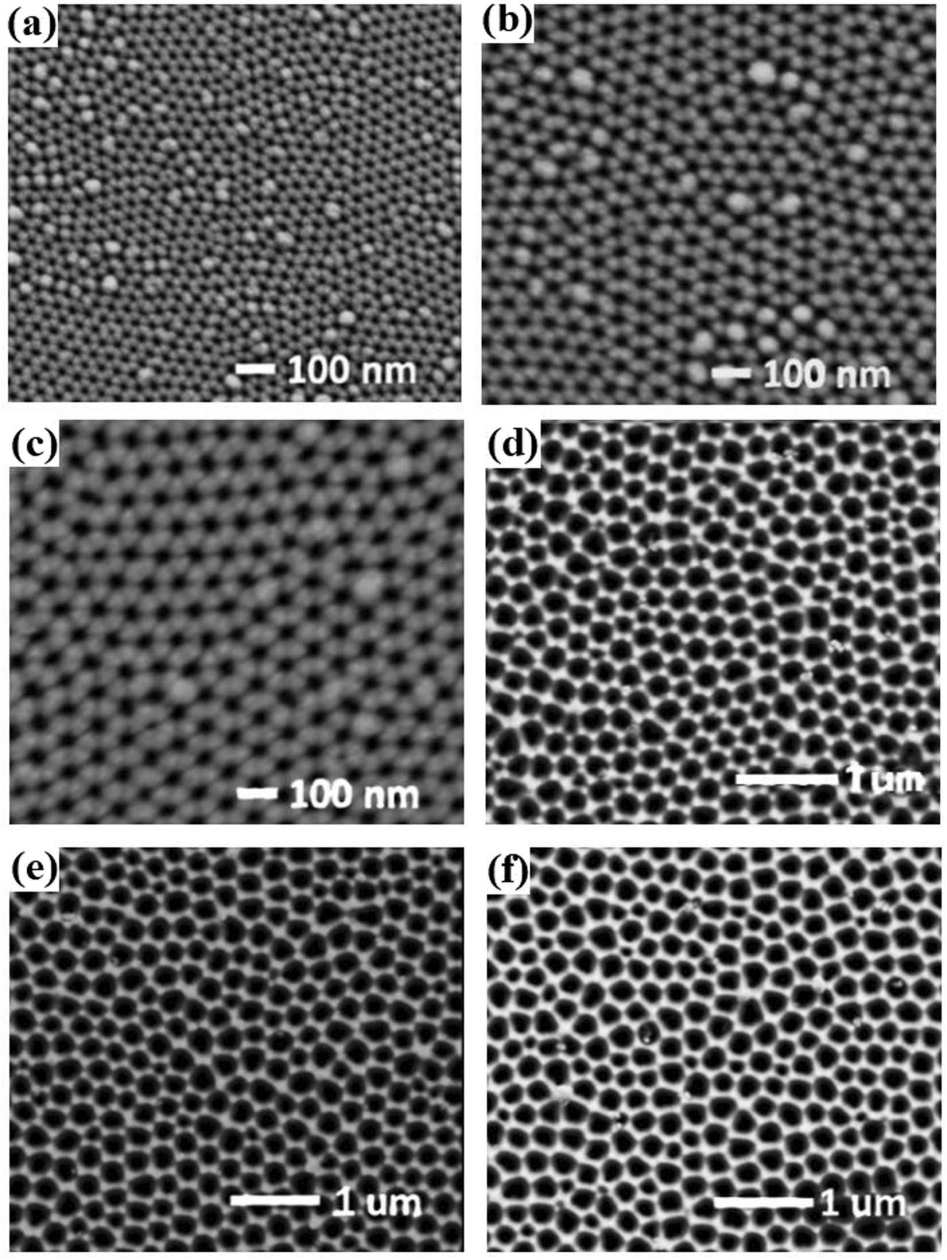
Top-viewed SEM images of multi-layered nanoporous arrayed films deposited on six AAO templates with different geometries (**a**) S = 110 nm, D = 40 nm, (**b**) S = 110 nm, D = 60 nm, (**c**) S = 110 nm, D = 100 nm, (**d**) S = 450 nm, D = 130 nm, (**e**) S = 450 nm, D = 160 nm and (**f**) S = 450 nm, D = 200 nm.

**Figure 2:**
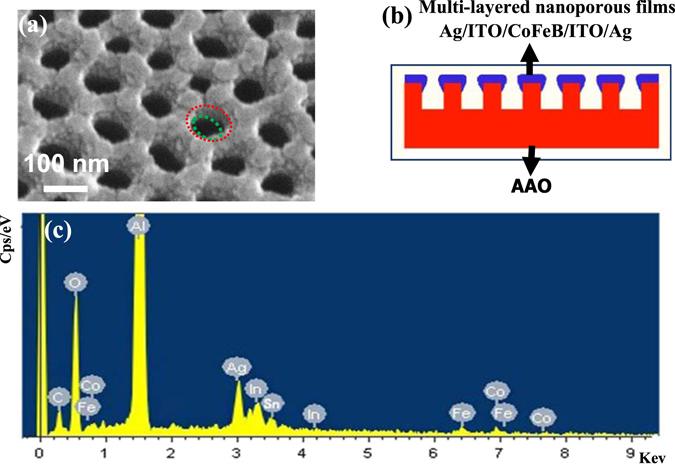
(**a**) Angle tilted magnified SEM images of Ag (5 nm)/ITO (10 nm)/CoFeB (10 nm)/ITO (10 nm)/Ag (5 nm) nanoporous films on S = 110 nm, D = 60 nm AAO template, (**b**) scheme of the cross-section of nanofunnel films and (**c**) EDS of one of multi-layered nanoporous films suggesting that these films have Ag, Co, Fe, Sn, In, Al and O elements.

Fig. 1 gives the typical top view SEM images of the already fabricated hybrid multi-layered nanoporous films. After deposition, the surface morphologies of the samples become more distinct and comparable with the changes of the AAO pore diameter (D) and inter-spacing (S). From the local-magnified angle-tilted SEM image (schemed in the Fig. 2a) of sample (b) (S = 110 nm, D = 60 nm), it is clearly noticed that most holes approximately present a nanofunnel morphology (the red circle is larger than the green one). A simplified nanostructure is sketched in Fig. 2b. EDS (Fig. 2c) confirms the existence of Ag, Co, Fe, In, Sn and O elements in the films and they originate from the deposited Ag layer, CoFeB layer and ITO layer^36, 100^ respectively.

### Magneto-Optical Kerr Rotation and Magnetic Domain

**Figure 3:**
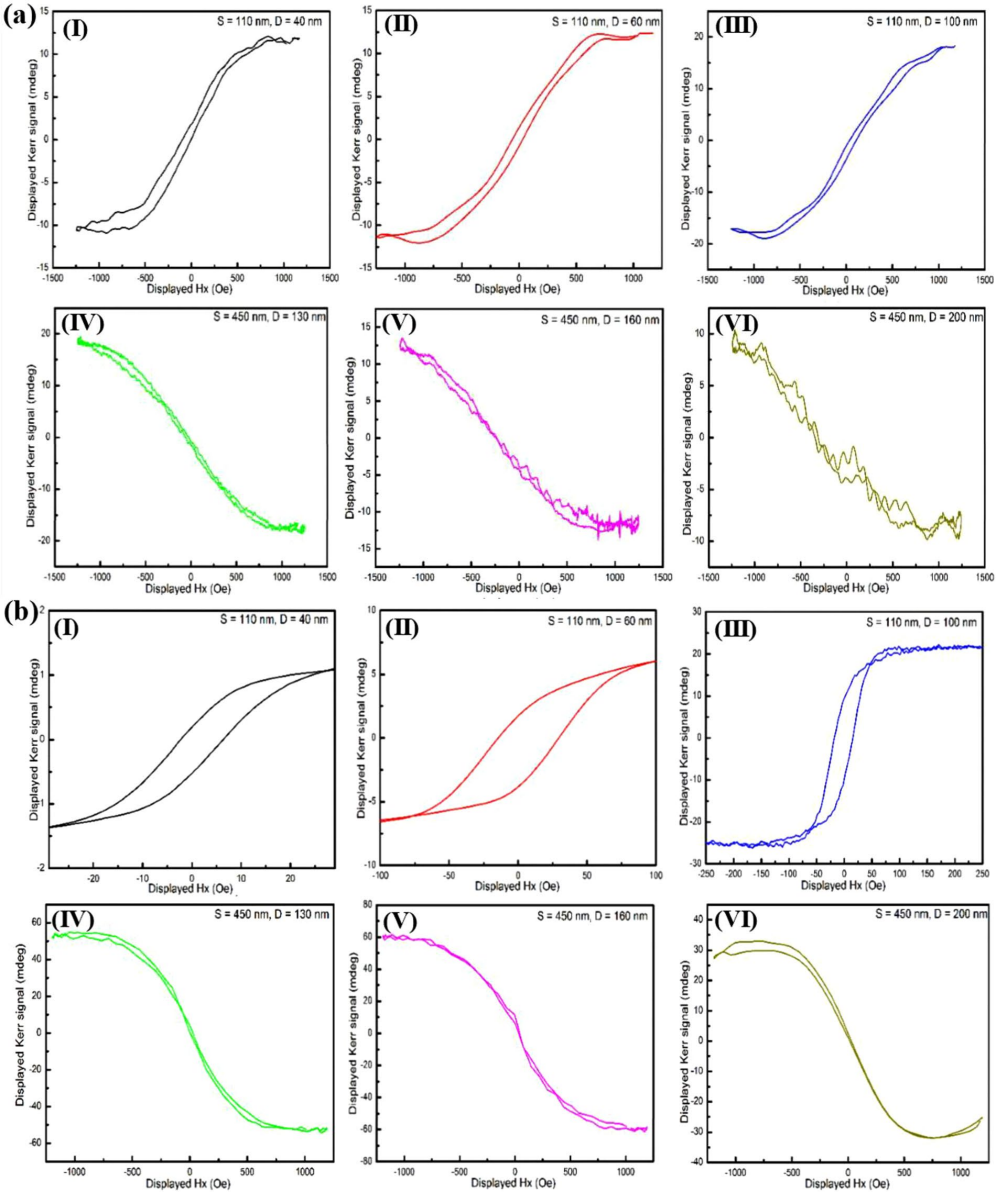
(**a**) P-MOKE and (**b**) L-MOKE loops of hybrid multi-layered nanoporous arrayed films deposited on six AAO templates with different geometries. (I) S = 110 nm, D = 40 nm; (II) S = 110 nm, D = 60 nm; (III) S = 110 nm, D = 100 nm; (IV) S = 450 nm, D = 130 nm; (V) S = 450 nm, D = 160 nm and (VI) S = 450 nm, D = 200 nm.

Fig. 3a shows the polar magneto-optical Kerr effect (P-MOKE) hysteresis loops of the prepared six nanoporous samples. At the first glance, it is evidently verified that an anomalous inversed Kerr hysteresis is observed by modifying the AAO porous film geometries (pore diameter and inter-spacing). Similarly, the sign inversion (Fig. 3b) is also detected in the longitudinal magneto-optical Kerr effect (L-MOKE) configuration simultaneously. It has been clarified that the sign of the MOKE is defined by both incoming and reflecting polarization of light, and a clockwise or an anticlockwise axis determines the sign change^50^. However, when the same incoming polarization and the same axis of the polarization ellipse viewed along the reflected direction are used, the inversion information in both P-MOKE and L-MOKE can be definitely observed.

The dramatic changes of the sign inversion can also be reflected by the direction rotation of typical magnetic domains (represented by the color mapping rotation, Fig. S1 in Supplementary Information) with the external applied magnetic field along the longitudinal direction of the samples. The first two samples ((I) S = 110 nm, D = 40 nm and (II) S = 110 nm, D = 60 nm) display an apparent color change rotation with the magnetic field changing from 1200 Oe (a), to 600 Oe (b), 0 Oe (c), −600 Oe (d), −1200 Oe (e), and back to -600 Oe (f), 0 Oe (g), 600 Oe (h) and 1200 Oe (i). However, as both the AAO pore diameters and inter-pore spacing are further increased (S = 450, D = 130 nm and S = 450 nm, D = 160 nm), directions of the magnetic domain distribution (as relatively indexed by the field intensity, or color change) distinctly show opposite behavior. Another result worth noting is that the magnetic domain distribution is not so distinctively exhibited when the AAO pore diameters are 100 nm (Fig. S1(III)) and 200 nm (Fig. S1(VI)), possibly due to the much reduced wall thickness among pores.

Therefore, under certain conditions, the sign of the MOKE loops in our proposal system is related to the pore diameter and inter-spacing. Thus, changing the AAO pore geometry allows us to tailor the MOKE sign. In order to unambiguously illustrate the physical mechanism of nanoporous structure related MO phenomena, the surface plasmon resonance property was investigated.

### Optical response

Fig. S2 in the Supplementary Information gives the absorbance of the pure AAO porous film (S = 110 nm, D = 60 nm) and the various films deposited on the same template. In this figure, the origin of oscillations observed in each spectrum is related to the optical modes of the Fabry-Pérot cavity^101, 102^. Furthermore, it is observed that when the Ag layer is coated on the surface of the AAO porous film, the absorbance intensity and amplitude of Fabry-Pérot interference resonances are greatly enhanced. It is well known that the noble metal Ag with a very high electrical conductivity can provide abundant free electrons, which can well couple with the electromagnetic (EM) field of light to generate the surface plasmon resonance. For the special designed hybrid multi-layered nanoporous structure (Ag (5 nm)/ITO (10 nm)/CoFeB (10 nm)/ITO (10 nm)/Ag (5 nm)) with a highly specific surface area, the resonance can be excited not only on the surface of the nanopore film but also localized around the pore side and the inner part of the pore.

**Figure 4:**
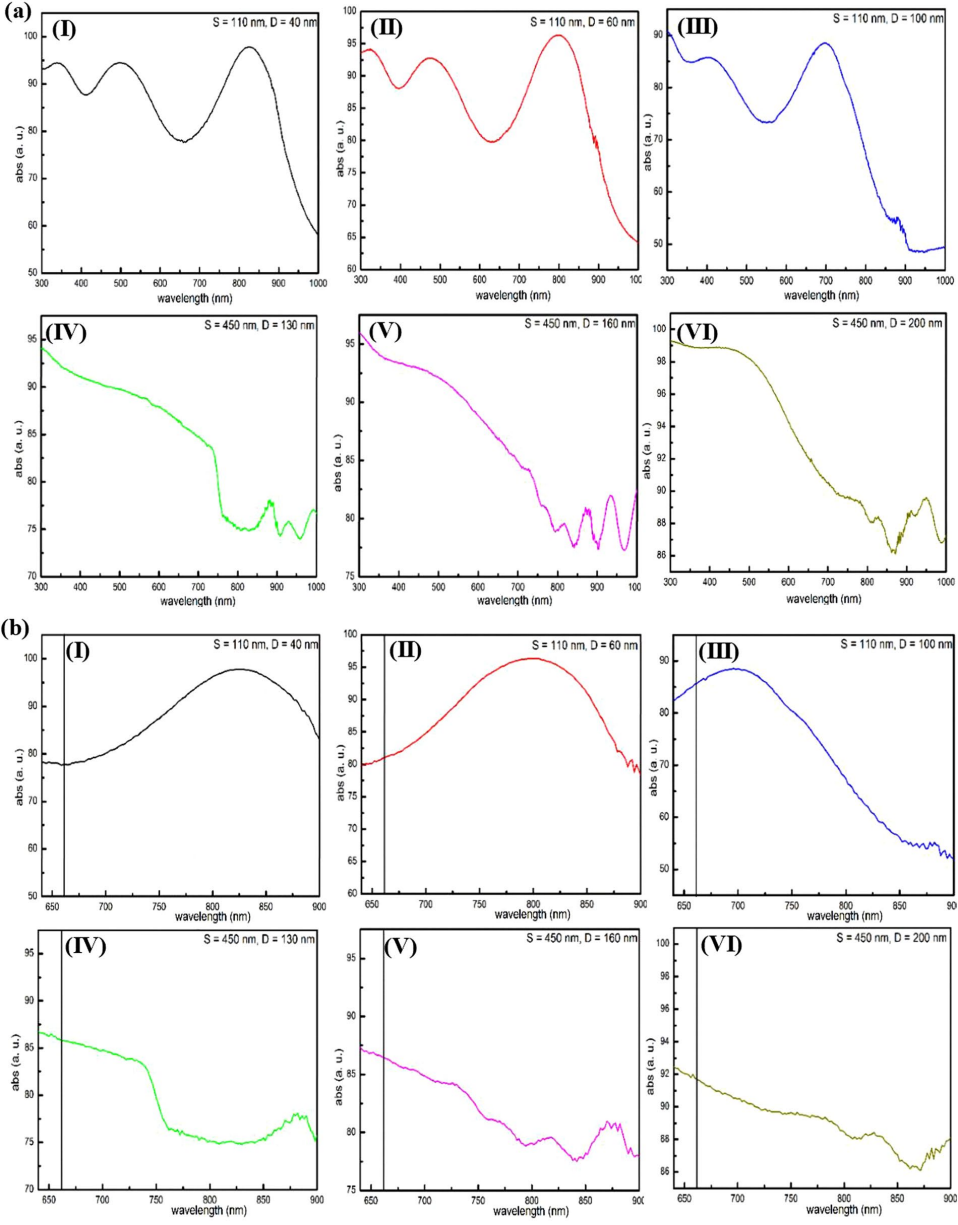
Absorbance intensity of hybrid multi-layered nanoporous arrayed films deposited on six AAO templates with different geometries. (I) S = 110 nm, D = 40 nm; (II) S = 110 nm, D = 60 nm; (III) S = 110 nm, D = 100 nm; (IV) S = 450 nm, D = 130 nm; (V) S = 450 nm, D = 160 nm and (VI) S = 450 nm, D = 200 nm. (**a**) Full range spectra. (**b**) Magnified local spectra with the vertical black line marks excitation at 660 nm.

Fig. 4a shows the optical absorbance of the prepared samples, whose resonances present an apparent Fabry-Pérot interference usually existing in the nanohole films^37^. Clearly, for the same inter-pore spacing 110 nm, the peaks exhibit a blue shift with the nanopore diameter increase from 40 nm, to 60 nm and to 100 nm at one optical oscillation period located between 640 nm and 900 nm (Fig. 4b). The wavelength of the laser light (660 nm) in our experiments is just in this wavelength range. Analysis of the Kerr hysteresis loops and the peak positions of the far-field absorbance intensity (Fig. 4b) indicates that there are the same signs when the peaks are at the same side of the given excitation line, while the signs are definitely inversed for those on the opposite sides (i.e., those with inter-spacing of 450 nm and diameter of 130 nm, 160 nm and 200 nm). From previous studies of magnetoplasmonics, oscillation shapes of the absorbance efficiency are directly related to the surface plasmon resonance^103–105^. Thus, it is concluded that the special MO phenomena are well related to the surface plasmon resonance which can be controlled by the designed nanoporous system.

## Discussion

The physical mechanism of the anomalous MOKE phenomena in the multi-layered nanoporous films can be unambiguously clarified. It is well known that the coupling of the transverse magnetic model (TM or p-polarization) and the transverse electric model (TE or s-polarization) can account for the sign inversion of the MOKE rotation^106, 107^. Taking P-MOKE as an example, when the TE electron oscillation mode in the noble metal Ag surface is excited owing to the introduced s-polarization light, due to the applied magnetic field, TM electron oscillation mode can be induced as a consequence of the spin-orbit (SO) coupling. The new hybrid oscillation mode has a strong effect on the Kerr information (i.e., Kerr rotation and ellipticity) and the phases between the two modes account for the sign of the Kerr rotation. It is well known that the tunable electron oscillations can be realized by the surface plasmon resonances, which are related to the designed nanostructure. Therefore, in this work, proper choosing the AAO porous film can control the surface plasmon resonance which has an effect on the electron oscillation modes. Furthermore, tunable sign inversion can be realized.

Besides the simplified physical mechanism of our result, we provide a clear expression of AAO geometry controlled effects on the MO response.

It is well known that when the magnetic field is applied to the surface of the sample, the permittivity tensor that contains nine complex components^51^ can be written in the following form:

1 $$\varepsilon =(\begin{array}{lll}{\varepsilon }_{xx} & i{\varepsilon }_{1} & i{\varepsilon }_{2}\\ -i{\varepsilon }_{1} & {\varepsilon }_{yy} & i{\varepsilon }_{3}\\ -i{\varepsilon }_{2} & -i{\varepsilon }_{3} & {\varepsilon }_{zz}\end{array})$$

where the diagonal component *ε*_*ii*_ (*i* = *x*, *y*, *z*) is the dielectric function of the non-magnetized material. This component does not depend on magnetization **M** when the first order magnetization is considered, and it has an effect only on the pure optical property. On the basis of the modified Drude model for the noble metal silver in our experiment, it can be expressed as

2 $${\varepsilon }_{ii}\approx {\varepsilon }_{\infty }-\frac{{\omega }_{p}^{2}}{{\omega }^{2}+i\gamma \omega }(i=x,y,z)$$

Here, *ω*_*p*_ is the Drude plasma frequency and $\gamma =\frac{1}{\tau }$ is the inverse electron relaxation time. The off-diagonal component *ε*_*i*_ (*i* = 1, 2, 3) is linearly dependent of magnetization^51^. For the different MOKE configurations, the control of the magneto-optical Kerr effect rotation originates from only one of the excited off-diagonal components *ε*_1_, *ε*_2_ and *ε*_3_ when the external magnetic field is applied along the *x*, *y* or *z* direction, which are related to the P-MOKE, transversal Kerr effect (T-MOKE) or L-MOKE, respectively.

**Figure 5:**
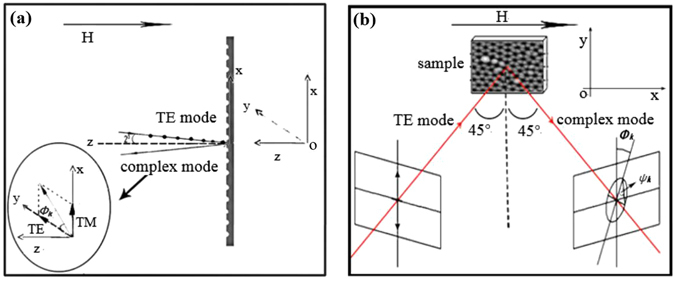
(**a**) P-MOKE and (**b**) L-MOKE measurement configurations.

For the P-MOKE in s-configurations (Fig. 5a), the off-diagonal term *ε*_1_ is activated by the magnetization samples along the *z* axis, and the new permittivity tensor can be described by the equation 3 in which all the other terms are equal to zero.

3 $${\varepsilon }_{p}=(\begin{array}{lll}{\varepsilon }_{xx} & i{\varepsilon }_{1} & 0\\ -i{\varepsilon }_{1} & {\varepsilon }_{yy} & 0\\ 0 & 0 & {\varepsilon }_{zz}\end{array})$$

When the TE electron oscillation mode with the amplitude *E*_*yy*_ is induced as a consequence of the incident light in the wavelength of 660 nm, the light induced complex dipole along the y axis can be written as a function of the polarization *α*_*yy*_, namely, *P*_*y*_ = *α*_*yy*_ *E*_*yy*_ (see refs 50, 51, 106, 108.). The complex dipole can be generated by the SO coupling that transfers the oscillations to the orthogonal direction.

4 $${P}_{x}={\alpha }_{xx}{E}_{so}={\alpha }_{xy}{E}_{yy}=\frac{-i{\varepsilon }_{1}{\alpha }_{xx}{\alpha }_{yy}}{{(\varepsilon -{\varepsilon }_{m})}^{2}}={\alpha }_{so}{\alpha }_{xx}{\alpha }_{yy}{E}_{yy}$$

In this expression, the constant *ε*_*m*_ is the dielectric function of the surrounding medium. The tuning of the phase and amplitude of the polarization can be achieved by the two orthogonal dipoles in the plane of the samples referring to our detecting system. The similar expression of the Kerr rotation in refs 1, 35 and 37 for nanoparticle arrays is used.

5 $${\theta }_{k}=\mathrm{Re}\,(\frac{{p}_{x}}{{p}_{y}})+\text{Im}\,(\frac{{p}_{x}}{{p}_{y}})i$$

In our investigation, we only focus on the Kerr rotation angle, named as *ϕ*_*k*_.

6 $$\begin{array}{rcl}{\varphi }_{k}^{p} & = & \mathrm{Re}(\frac{{p}_{x}}{{p}_{y}})=\mathrm{Re}(\frac{{\alpha }_{so}{\alpha }_{xx}{\alpha }_{yy}{E}_{yy}}{{\alpha }_{yy}{E}_{yy}})\\  & = & \mathrm{Re}({\alpha }_{so}{\alpha }_{xx})\\  & = & \mathrm{Re}({\alpha }_{so}^{0}{e}^{i\varphi ({\alpha }_{so})}{\alpha }_{xx}^{0}{e}^{i\varphi ({\alpha }_{xx})})\\  & = & \mathrm{Re}({\alpha }_{so}^{0}{\alpha }_{xx}^{0}{e}^{i(\varphi ({\alpha }_{so})+\varphi ({\alpha }_{xx}))})\\  & = & \mathrm{Re}({\alpha }_{so}^{0}{\alpha }_{xx}^{0}{e}^{i\varphi })\\  & = & \mathrm{Re}({\alpha }_{so}^{0}{\alpha }_{xx}^{0}\,\cos \,\varphi -i{\alpha }_{so}^{0}{\alpha }_{xx}^{0}\,\sin \,\varphi )\\  & = & {\alpha }_{so}^{0}{\alpha }_{xx}^{0}\,\cos \,\varphi \end{array}$$

The equation shows how the Kerr rotation ${\varphi }_{k}^{p}$ is related to the two phases of the intrinsic properties *α*_*so*_ and *α*_*xx*_ owing to the excitation along *x* axis in the s-configurations. Clearly, the exponential notation expressions (${\alpha }_{so}^{0}{e}^{i\varphi ({\alpha }_{so})}$ and ${\alpha }_{xx}^{0}{e}^{i\varphi ({\alpha }_{xx})}$) can definitely describe the combined phase *ϕ* between the two polarizations (*α*_*so*_ and *α*_*xx*_). Clearly, by selecting proper diameters and distances between two nanopores, the combined phase can be changed enough to produce a reversal sign.

However, for the L-MOKE in s-configuration (Fig. 5b), the off-diagonal term *ε*_3_ is activated by the magnetization along the *x* axis, and the new permittivity tensor can be described by the equation 7 in which all the other terms are equal to zero.

7 $${\varepsilon }_{L}=(\begin{array}{ccc}{\varepsilon }_{xx} & 0 & 0\\ 0 & {\varepsilon }_{yy} & i{\varepsilon }_{3}\\ 0 & -i{\varepsilon }_{3} & {\varepsilon }_{zz}\end{array})$$

Through the SO coupling, the light induced dipole *P*_*y*_ generates *P*_*z*_.

8 $${P}_{z}={\alpha }_{yy}{E}_{so}={\alpha }_{zy}{E}_{yy}=\frac{-i{\varepsilon }_{3}{\alpha }_{zz}{\alpha }_{yy}}{{(\varepsilon -{\varepsilon }_{m})}^{2}}{E}_{yy}={\alpha }_{so}{\alpha }_{zz}{\alpha }_{yy}{E}_{yy}$$

And the Kerr rotation ${\varphi }_{k}^{L}$ in the L-MOKE configuration can be expressed as:

9 $$\begin{array}{rcl}{\varphi }_{k}^{L} & = & \mathrm{Re}(\frac{{p}_{z}}{{p}_{y}})=\mathrm{Re}(\frac{{\alpha }_{so}{\alpha }_{zz}{\alpha }_{yy}{E}_{yy}}{{\alpha }_{yy}{E}_{yy}})\\  & = & \mathrm{Re}({\alpha }_{so}{\alpha }_{zz})\\  & = & \mathrm{Re}({\alpha }_{so}^{0}{e}^{i\varphi ({\alpha }_{so})}{\alpha }_{zz}^{0}{e}^{i\varphi ({\alpha }_{zz})})\\  & = & \mathrm{Re}({\alpha }_{so}^{0}{\alpha }_{zz}^{0}{e}^{i(\varphi ({\alpha }_{so})+\varphi ({\alpha }_{zz}))})\\  & = & \mathrm{Re}({\alpha }_{so}^{0}{\alpha }_{zz}^{0}{e}^{i\varphi })\\  & = & \mathrm{Re}({\alpha }_{so}^{0}{\alpha }_{zz}^{0}\,\cos \,\varphi -i{\alpha }_{so}^{0}{\alpha }_{zz}^{0}\,\sin \,\varphi )\\  & = & {\alpha }_{so}^{0}{\alpha }_{zz}^{0}\,\cos \,\varphi \end{array}$$

Different from the P-MOKE configuration, ${\varphi }_{k}^{L}$ is related to the two phases of the intrinsic properties *α*_*so*_ and *α*_*zz*_ due to the excitation along *z* axis in the s-configurations.

## Conclusion

In this work, we have presented an exhaustive analysis about correlation of the hybrid multi-layered nanoporous system and the inversion sign of the Kerr hysteresis. The analysis confirms that the phases of the TM and TE electron oscillation modes account for the sign inversion of the MOKE rotation. The electron oscillation modes can be tuned at will by the surface plasmon resonance which is controlled by choosing an AAO porous film with proper pore diameter and inter-pore spacing.

The study broadens the understanding of MOKE in the nanoporous magnetoplasmonic systems. It is an opportunity to allow a more precise design of nanostructured optical and magneto-optical devices for future biotechnological applications and magneto-optical sensors.

## Methods

### Fabrication of Hybrid Multi-layered Nanoporous Films with Controlled AAO Substrates

Hybrid multi-layered films Ag (5 nm)/ITO (10 nm)/CoFeB (10 nm)/ITO (10 nm)/Ag (5 nm) were fabricated on the prepared six different AAO substrates, respectively, by a multi-step magnetron sputtering deposition process at room temperature^36, 37^. Noticing that the noble metallic Ag and conductive indium tin oxides (ITO) targets were sputtered in a 15 mTorr Ar gas atmosphere using the direct current (dc) sputtering apparatus (MSP-300C, WeiNa Chuangshi Inc.) with the power of 50 W. The ferromagnetic CoFeB layer was sputtered in a 0.75 mTorr Ar gas atmosphere using the radio frequency (rf) sputtering apparatus with the power of 100 W.

### Characterization of the Structure and Composition

Scanning electron microscope (SEM) coupled with energy-dispersive X-ray spectroscopy (EDS) was used to characterize the top morphologies , angle tilted magnified surface information and compositions of the multi-layered nanoporous films.

### Characterization of Magneto-optical Kerr Rotation and Magnetic Domain

The magneto-optical activity and the information of the magnetic domains of the prepared samples were investigated by the NanoMOKE-3 instrument (Durham Magneto Optics Ltd), a high-performance magneto-optical magnetometer and Kerr microscope, capable of both laser magnetometry and video Kerr microscopy.

With 660 nm monochromatic s-polarization light (incident angle 2°) and proper choice of a continuously changed external applied magnetic field **H** whose direction is perpendicular to the sample’s surface, the polar magneto-optical Kerr effect (P-MOKE) with the magnetic field ranged from −1250 Oe to +1250 Oe with a ramp of 2.061 Oe/step was characterized. This experiment configuration was sketched in Fig. 5a.

As shown in Fig. 5b, when the direction of the magnetic field was parallel to the surface and the s-polarization light’s incident angle was changed to 45°, the longitudinal magneto-optical Kerr effect (L-MOKE) information can be collected. In this experiment, similarly to the P-MOKE configuration, the excitation light wavelength was also 660 nm.

### Characterization of optical response

In order to construct a reasonable model to interpret the special MO phenomena in hybridization of nanostructures and find the optical resonance position defined by the amplitude and the phase of the dipolar surface plasmon resonance (SPR) directly^109, 110^, far-field absorbance was evaluated and analyzed by the conventional UV-Vis-NIR spectrophotometer (UV 3600, 190 nm-3500 nm) from 300 nm to 1000 nm at room temperature.

## Electronic supplementary material

Supplementary Information
