# Supplementary material for: Tunable Magneto-Optical Kerr Effects of Nanoporous Thin Films
Source: Sci Rep. 2017 Jun 6;7:2888. doi: 10.1038/s41598-017-03241-7 (PMC5460283; doi:10.1038/s41598-017-03241-7)

Supplementary Information for

Tunable Magneto-Optical Kerr Effects of Nanoporous Thin Films

Weiwei Zhang^1^, Jianjun Li^1^, Xiaokun Ding^2^, Philippe Pernod^2^, Nicolas Tiercelin^2^, Yujun Song^1^*

^1^ Department of Applied Physics, Center for Modern Physics Technology, Beijing Key Laboratory for Magneto-Photoelectrical Composite and Interface Science, University of Science and Technology Beijing, Beijing 100083, China.

*Email: songyj@ustb.edu.cn

^2^Univ. Lille, CNRS, Centrale Lille, ISEN, Univ. Valenciennes, UMR 8520-IEMN-LIA LICS, F-59000 Lille, France.

**Abstract:** Magnetoplasmonics, combining magnetic and plasmonic functions, have attracted increasing attention owing to their unique magnetic and optical properties in various nano-architectures. In this work, Ag, CoFeB and ITO layers are fabricated on anodic aluminum oxide (AAO) porous films to form hybrid multi-layered nanoporous thin films by magneto-sputtering deposition process. The designed nanostructure supports localized surface plasmon resonance (LSPR) and tunable magneto-optical (MO) activity, namely, the sign inversion, which can be controlled by AAO porous film geometry (pore diameter and inter-pore spacing) flexibly. The physical mechanism of this special MO phenomena is further analyzed and discussed by the correlation of Kerr rotation and electronic oscillations controlled by the surface plasmon resonance that is related to the nanoporous structure.

**Figure S1.** Magnetic field dependent magnetic domains change of different pore diameters (D) and inter-pore spacing (S) along the longitudinal direction at different fields representing by color maping change with the magnetic field at (a) 1200 Oe. (b) 600 Oe. (c) 0 Oe. (d) -600 Oe. (d) -1200 Oe. (f) -600 Oe. (g) 0 Oe. (h) 600 Oe. (i) 1200 Oe. (I) D = 40 nm, S = 110 nm; (II) D = 60 nm, S = 110 nm; (III) D = 100 nm, S = 110 nm; (IV) D = 130 nm, S = 450 nm; (V) D = 160 nm, S = 450 nm; (VI) D = 200 nm, S = 450 nm.


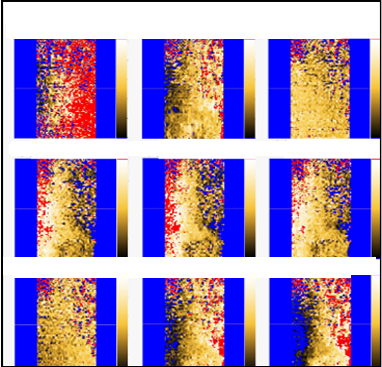


D = 40 nm, S = 110 nm

**(I)**

**(a)**

**(b)**

**(c)**

**(d)**

**(e)**

**(f)**

**(g)**

**(h)**

**(i)**

1200 oe

600 oe

0 oe

-600 oe

-1200 oe

-600 oe

0 oe

600 oe

1200 oe


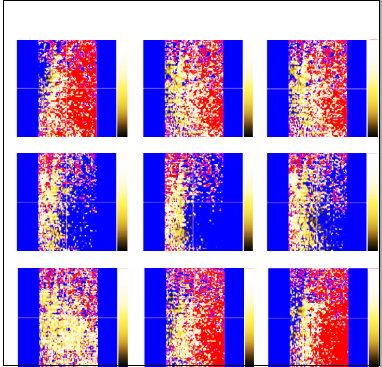


**(II)**

**(b)**

**(c)**

**(d)**

**(e)**

**(f)**

**(g)**

**(h)**

**(i)**

**(a)**

D = 60 nm, S = 110 nm

1200 oe

600 oe

0 oe

-600 oe

-1200 oe

-600 oe

0 oe

600 oe

1200 oe


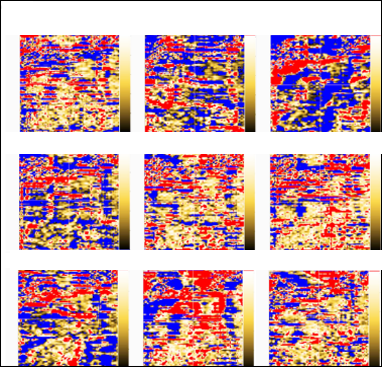


**(III)**

**(b)**

**(c)**

**(d)**

**(e)**

**(f)**

**(g)**

**(h)**

**(i)**

D = 100 nm, S = 110 nm

1200 oe

600 oe

0 oe

-600 oe

-1200 oe

1200 oe

-600 oe

600 oe

0 oe


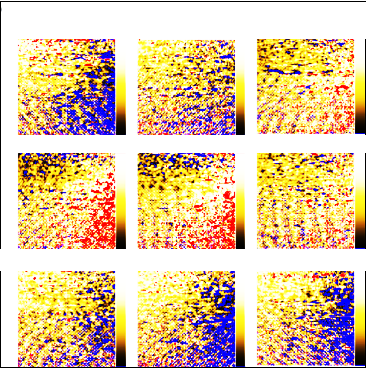


**(IV)**

D = 130 nm, S = 450 nm

**(b)**

**(c)**

**(d)**

**(e)**

**(f)**

**(g)**

**(h)**

**(i)**

1200 oe

600 oe

0 oe

1200 oe

600 oe

0 oe

-600 oe

-600 oe

-1200 oe


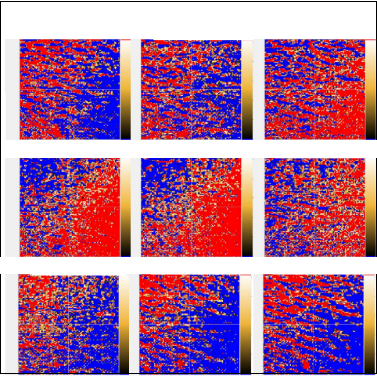


**(V)**

**(b)**

**(c)**

**(d)**

**(e)**

**(f)**

**(g)**

**(h)**

1200 oe

D = 160 nm, S = 450 nm

600 oe

0 oe

-600 oe

-1200 oe

-600 oe

600 oe

0 oe

1200 oe


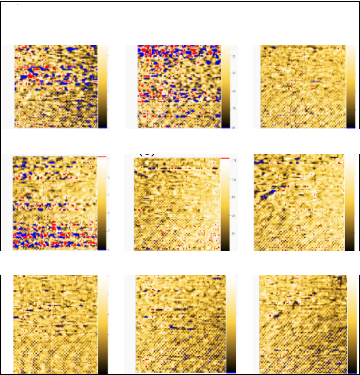


**(VI)**

**(d)**

**(g)**

**(b)**

**(e)**

**(h)**

**(c)**

**(f)**

**(i)**

D = 200 nm, S = 450 nm

1200 oe

600 oe

0 oe

-600 oe

-1200 oe

-600 oe

0 oe

600 oe

1200 oe

**Figure S2.** UV-vis absorbance of multi- layered films: AAO template (S =110 nm D = 60 nm) (black); Pure Ag (10 nm) (red); Conductive ITO (10 nm) (blue); CoFeB (10 nm) (magenta); Hybrid multi-layered films: Ag (5 nm)/CoFeB (10 nm)/Ag (5 nm) (green); Hybrid multi-layered films: Ag (5 nm)/ITO (10 nm)/CoFeB (10 nm)/ITO (10 nm)/Ag (5 nm) (dark yellow).


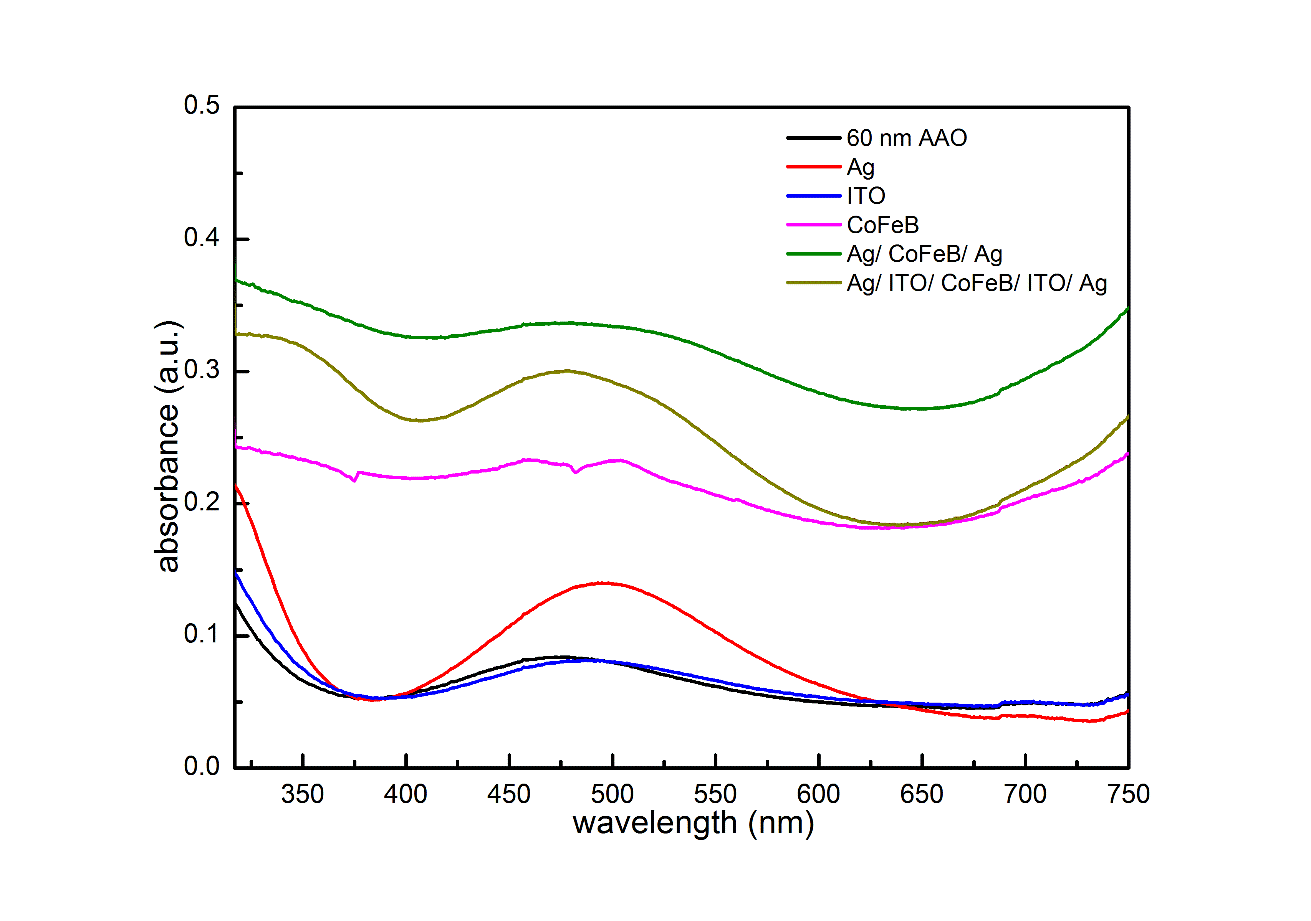

Supplement: Supplementary file 1 — Supplementary Information [file 41598_2017_3241_MOESM1_ESM.doc]
